# Clinical manifestation and genetic analysis in Chinese early onset X‐linked retinoschisis

**DOI:** 10.1002/mgg3.1421

**Published:** 2020-07-21

**Authors:** Li Huang, Limei Sun, Zhirong Wang, Chonglin Chen, Panfeng Wang, Wenmin Sun, Xiaoling Luo, Xiaoyan Ding

**Affiliations:** ^1^ State Key Laboratory of Ophthalmology Zhongshan Ophthalmic Center Sun Yat‐sen University Guangzhou China

**Keywords:** complications, early onset, rare phenotypes, *RS1*, X‐linked retinoschisis

## Abstract

**Background:**

X‐linked retinoschisis (XLRS) is one type of retinal dystrophy leading to the schisis of the neural retina and causing reduced visual acuity. The study aimed to investigate the clinical manifestations and retinoschisin 1 (*RS1*) mutations in Chinese patients with early onset XLRS.

**Methods:**

Thirty‐eight probands with early onset XLRS were recruited, comprehensive ophthalmic examination was performed. A targeted gene panel was used to test the *RS1* mutations.

**Results:**

All probands had *RS1* hemizygous mutations including 16 known and 14 novel mutations. The median onset age was 2 years old (range 0.1–6 years). Probands with onset age ≤1 years. had more complications (retinal detachment and vitreous hemorrhage,* p* < 0.001), more mutations outside the discoidin domain and more non‐frameshift mutations than probands with onset age >1 years. Macular and peripheral involvement was present in 77.27% of probands, and inner and outer nuclear layer splitting were present in 53.57% of probands. Electroretinography showed an electronegative waveform. The relatively rare phenotypes of lamellar macular hole and macular hole were present in a unilateral eye in three probands.

**Conclusion:**

In conclusion, the early onset XLRS developed more severe complications which need close monitoring and clinical manifestations illustrated here may facilitate the early diagnosis of retinoschisis.

## Graphical abstract

The median onset age was 2 years old (years, range 0.1–6 years). Probands with onset age (≤1 years) had more complications (retinal detachment and vitreous hemorrhage, *p* < 0.001), more mutations outside the discoidin domain and more non‐frameshift mutations than probands with onset age (>1 years). Macular and peripheral involvement was present in 77.27% of probands, and inner and outer nuclear layer splitting were present in 53.57% of probands.

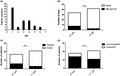

## 1 INTRODUCTION

X‐linked retinoschisis (XLRS; MIM 312700) is one type of retinal dystrophy that leads to the schisis (splitting) of the neural retina, causing reduced visual acuity. It is the leading cause of macular degeneration in male children with an estimated prevalence ranging between 1:5,000 and 1:20,000 (George, Yates, & Moore, 1995; Molday, Kellner, & Weber, 2012). The typical clinical feature of XLRS is a spoke‐wheel pattern foveal schisis that is associated with a peripheral schisis in about 50% of the affected patients. Foveal involvement has been reported in all affected patients, with mildly to severely reduced visual acuity (George et al., 1995; Tantri et al., 2004). An absent or severely reduced b‐wave found via electroretinography (ERG) is typical for XLRS (Tantri et al., 2004). Spectral‐domain optical coherence tomography (OCT) is the major diagnostic technique for identifying XLRS. However, because of the inclusion of the infants and preschool children, it is difficult to perform these examinations on the young children.

The retinoschisin 1 (*RS1*) gene is located on the X chromosome. It contains six exons that encode retinoschisin, express exclusively in the retina and function as an octamer. The gene is also implicated in cell‐cell adhesion and interactions (Sikkink, Biswas, Parry, Stanga, & Trump, 2007). RS1 protein contains three functional domains, including a signal sequence (exons 1 and 2), retinoschisin domain (exon 3), and discoidin domain (exons 4–6) (Sauer et al., 1997), which are important during retinal development (The Retinoschisis Consortium, 1998). To date, more than 240 unique mutations in the *RS1* gene have been reported as associated with retinoschisis (RS) phenotypes (Human Gene Mutation Database [HGMD], http://www.hgmd.cf.ac.uk/ac/index.php), including 154 missense and nonsense mutations and 23 splicing mutations, with the remaining as small deletions, small insertions, and gross deletions. A great proportion of the mutations are located in the discoidin domain (HGMD).

The clinical manifestations of XLRS exhibit a large degree of clinical variability (Apaolaza et al., 2016; Xiao et al., 2016). Previous studies have failed to identify a genotype‐phenotype correlation because of this variability in clinical features. Clinical variability can also lead to misdiagnosis, especially in patients with early onset like XLRS. In this study, we attempted to illustrate the clinical manifestations of early onset XLRS in a Chinese population.

## 2 METHODS

### 2.1 Participants

The data from 38 unrelated families with XLRS were collected from the retinal department at Zhongshan Ophthalmic Center, Guangzhou, China, from February 2016 to December 2018. The recruitment criteria included a clinical diagnosis of RS conformed by fundus photography (FP) or OCT and a pathogenic mutation in the *RS1* gene as well as a disease onset age of less than 6 years old.

### 2.2 Editorial policies and ethical considerations

This study was approved by the Institutional Review Board of the Zhongshan Ophthalmic Center and was performed in accordance with the guidelines described in the Declaration of Helsinki. Informed consent was obtained from all participants or their guardians prior to the collection of the clinical data and genomic samples. The genomic DNA was extracted from the leukocytes of venous blood using previously reported methods (Tang et al., 2016).

### 2.3 Sequencing

The genetic testing was performed using a clinical panel targeting genes that are common causes of inherited eye diseases. The targeted resequencing was performed using the Illumina MiSeq platform (Illumina, Madison, WI, USA) with a SeqCap EZ Library SR kit (Roche NimbleGen Roche, Inc., Madison, WI, USA), Illumina MiSeq v2 kit and Illumina PhiX Control v3 kit. The average sequencing depth was set to 100‐fold. Strand NGS software version 2.0 (Strand Scientific Intelligence Inc.) was used to set the sequencing reads to University of California Santa Cruz hg19. The variation annotation was conducted using single nucleotide polymorphism (SNP) effects analysis software. SIFT, Polyphen2_HDIV, Polyphen2_HVAR, LRT, MutationTaster, MutationAssessor, FATHMM, MetaSVM, and MetaLR were used for the bioinformatics analysis. Sanger sequencing was used to verify the mutations and perform a segregation analyses on available family members. All the mutation nomenclature followed Human Genome Variation Society (HGVS) guidelines for mutation description (http://www.hgvs.org). Allele frequencies of the variants detected in this study were confirmed by The Exome Aggregation Consortium (ExAC; http://exac.broadinstitute.org).

### 2.4 Clinical examination

The clinical data collected included the age, gender, onset age, first symptom, and family history of subjects. A complete ophthalmic examination, including a best corrected visual acuity (BCVA) measurement, slit‐lamp examination, and fundus biomicroscopy, was performed in each patient and their available family members. FP, ERG, and OCT were done with parental consent as required. FP was performed with an FF450 fundus camera (Zeiss, Oberkochen, Germany), scanning laser ophthalmoscope (SLO, Nidek F‐10, Gamagori, Japan), or RetCam (Clarity Medical Systems, Pleasanton, CA, USA). ERG was performed with a RETeval (LKC Technologies, Gaithersburg, MD, USA). OCT was performed with a Spectralis HRA (Heidelberg Engineering, Heidelberg, Germany).

### 2.5 Statistics

The statistics was performed using the IBM SPSS Statistics 21.

## 3 RESULTS

### 3.1 Mutations

**Figure 1:**
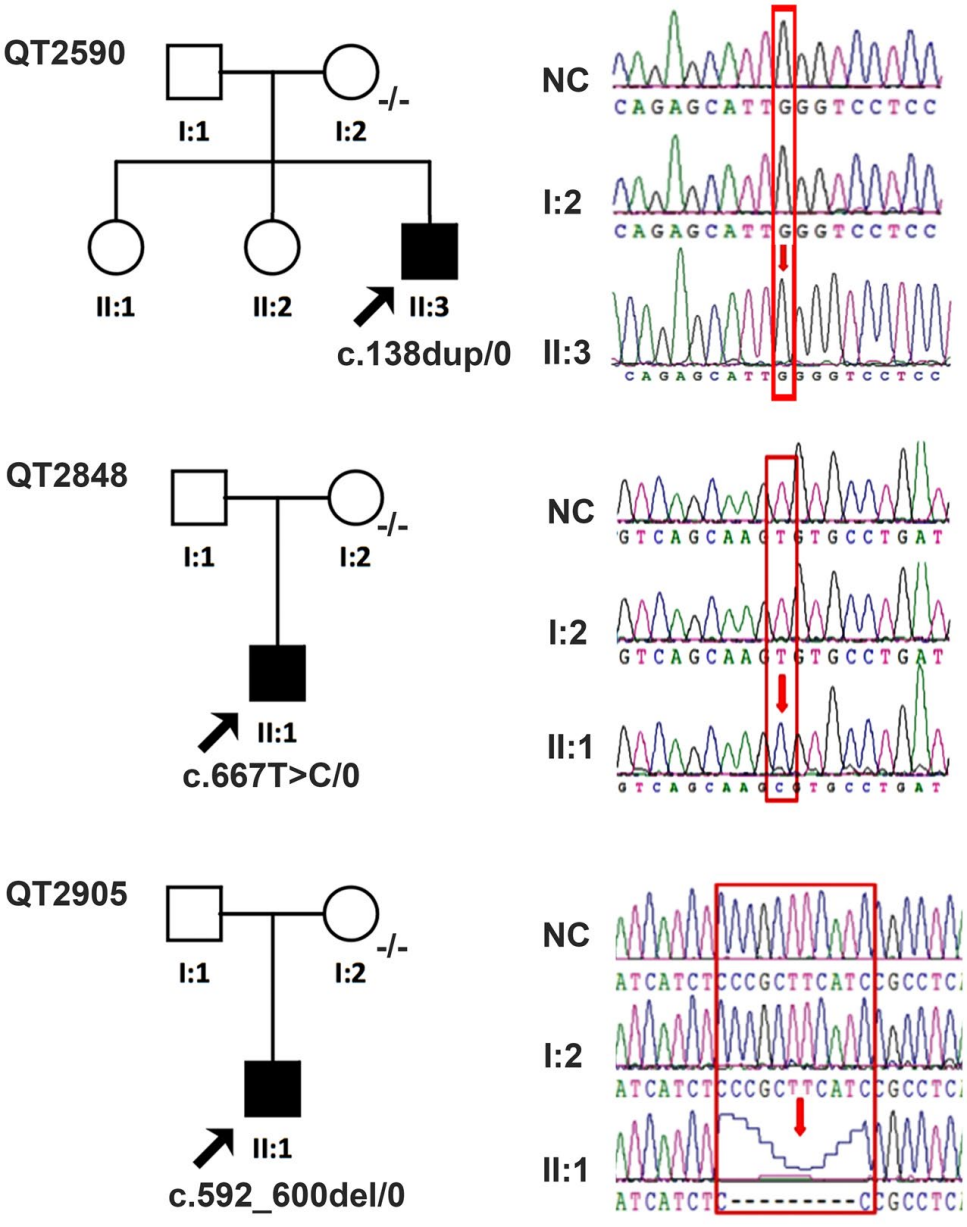
The pedigrees and sequence chromatography of a family with de novo mutations. Proband QT2590 with a hemizygous c.138dup mutation, proband QT2848 with c.667T>C mutation and QT2905 with a c.592_600del mutation. NC, normal control

**Figure 2:**
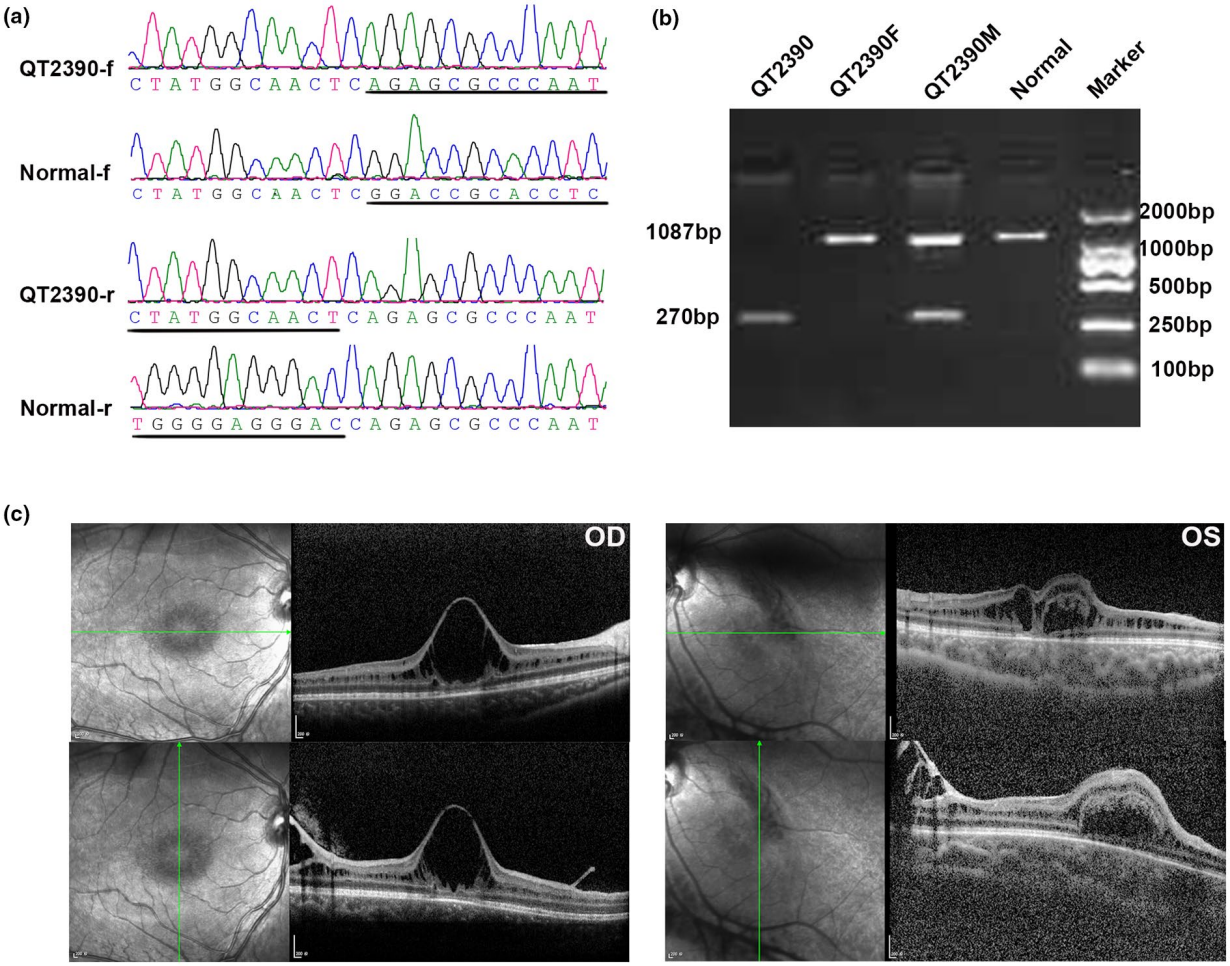
Sequence chromatography, segregation analysis and OCT of proband QT2390 with the c.540_675+682del mutation. (a) Sequence chromatography: ‐f stands for sequencing from the forward direction and ‐r stands for sequencing from the reverse direction. (b) Agarose gel electrophoresis image: the amplicons from the father and normal control were 1,087 bp each, the amplicons from the mother harboring a heterozygous c.540_675+682del mutation were 1,087 and 270 bp, and the amplicon from the proband harboring a hemizygous c.540_675+682del mutation was 270 bp. (c) OCT of QT2390 showing the inner nuclear layer splitting of the retina

There were 38 probands included in this study with a pathogenic mutation detected in the *RS1* gene, including 16 known and 14 novel mutations (Table 1) (Gehrig et al., 1999; Hiriyanna et al., 1999; Hotta et al., 1998; Molday et al., 2012; Sato, Oshika, Kaji, & Nose, 2003; The Retinoschisis Consortium, 1998; Yu, Li, Li, & Zhang, 2001). All of the mutations were hemizygous and could be verified via Sanger sequencing. Segregation analyses were performed in 35 probands and the available family members, of whom 32 unaffected mothers had a heterozygous *RS1* mutation. De novo mutations were detected in three families: c.138dup (p.Asn47Glnfs) in proband QT2590, c.667T>C (p.Cys223Arg) in proband QT2848 and c.592_600del (p.Phe198_Arg200del) in proband QT2905, all of which were absent in their mothers (Figure 1). Proband QT3526 had two *RS1* mutations c.193T>C (p.Tyr65His) and c.185‐1G>C as did his unaffected mother. Among the 38 probands, the mutation proportions were missense (25/39, 64.10%), small indels (6/39, 15.38%), nonsense (4/39, 10.25%), frame shift (3/39, 7.69%), and gross deletion (1/39, 2.56%). The mutant alleles were either absent from the ExAC or had a low allele frequency of less than 1/87,544 (Table 1). Ten mutations (25.64%) were detected outside the discoidin domain, and 29 mutations (74.36%) were located within the discoidin domain. The known mutations c.214G>A (p.Glu72Lys), c.305G>A (p.Arg102Gln), and c.579dup (p.Ile194fs) were detected in three (7.89%), four (10.53%), and three (10.53%) probands, respectively, and were considered to be the mutation hot spots of *RS1*. The novel gross deletion (c.540_675+682del) was verified via Sanger sequencing from forward and the reverse directions (Figure 2a). The segregation analysis was confirmed by agarose gel electrophoresis (Figure 2b). The proband QT3526 had two mutations c.193T>C (p.Tyr65His) and c.185‐1G>C in one allele inherited from his mother.

**Table 1:** *RS1* mutations detected in this study

Family ID	Location	DNA change	Amino acid change	Bioinformatics	ExAC	States	State of mother	Reference
QT2172	18674782	c.175T>G	p.Cys59Gly	probably damaging	0	Hemi	hetero	Novel
QT2173	18660152	c.647T>C	p.Leu216Pro	probably damaging	0	Hemi	NA	The Retinoschisis Consortium (1998)
QT2294	18665332	c.305G>A	p.Arg102Gln	probably damaging	0	Hemi	hetero	The Retinoschisis Consortium (1998)
QT2312	18660191	c.608C>T	p.Pro203Leu	probably damaging	0	Hemi	hetero	The Retinoschisis Consortium (1998)
QT2325	18660212	c.587C>T	p.Ser196Phe	probably damaging	0	Hemi	hetero	Novel
QT2329	18690163	c.26T>A	p.Leu9*	–	0	Hemi	hetero	Novel
QT2347	18662681	c.391A>C	p.Lys131Gln	probably damaging	0	Hemi	hetero	Novel
QT2361	18665361	c.276G>C	p.Trp92Cys	probably damaging	0	Hemi	hetero	Hotta et al. (1998)
QT2390	18660256	c.540_675+682del	–	splicing change	0	Hemi	hetero	Novel
F1	18665332	c.305G>A	p.Arg102Gln	probably damaging	0	Hemi	hetero	The Retinoschisis Consortium (1998)
F2	18660156	c.643G>A	p.Glu215Lys	probably damaging	0	Hemi	Hetero	Gehrig et al., 1999
F3	18665332	c.305G>A	p.Arg102Gln	probably damaging	0	Hemi	Hetero	The Retinoschisis Consortium (1998)
F4	18674859	c.98G>A	p.Trp33*	–	0	Hemi	Hetero	Chen et al. (2014)
F5	18665423	c.214G>A	p.Glu72Lys	probably damaging	1/87523	Hemi	Hetero	The Retinoschisis Consortium (1998)
F6	18665332	c.305G>A	p.Arg102Gln	probably damaging	0	Hemi	Hetero	The Retinoschisis Consortium (1998)
QT2462	18660224	c.575C>A	p.Pro192His	probably damaging	0	Hemi	Hetero	Novel
QT2571	18660162	c.637C>T	p.Arg213Trp	probably damaging	0	Hemi	Hetero	The Retinoschisis Consortium (1998)
QT2590	18674818	c.138dup	p.Asn47Glnfs	frameshift	0	Hemi	normal	Novel
QT2650	18665423	c.214G>A	p.Glu72Lys	probably damaging	1/87523	Hemi	Hetero	Yu et al. (2001)
QT2681	18690132	c.52+5G>A	–	splicing change	1/87759	Hemi	Hetero	Novel
QT2905	18660203	c.592_600del	p.Phe198_Arg200del	–	0	Hemi	normal	Novel
QT2765	18660219	c.579dup	p.Ile194fs	frameshift	0	Hemi	Hetero	The Retinoschisis Consortium (1998)
QT2848	18660132	c.667T>C	p.Cys223Arg	probably damaging	0	Hemi	normal	Hiriyanna et al. (1999)
QT3065	18660212	c.587C>T	p.Ser196Phe	probably damaging	0	Hemi	Hetero	Novel
QT3046	18665423	c.214G>C	p.Glu72Gln	probably damaging	1/87523	Hemi	Hetero	The Retinoschisis Consortium (1998)
QT3032	18660219	c.579dup	p.Ile194fs	frameshift	0	Hemi	Hetero	The Retinoschisis Consortium (1998)
QT2337	18665450	c.187T>C	p.Cys63Arg	probably damaging	0	Hemi	Hetero	Novel
QT3042	18660209	c.590G>A	p.Arg197His	probably damaging	0	Hemi	Hetero	The Retinoschisis Consortium (1998)
QT2931	18662549	c.522+1G>A	–	splicing change	0	Hemi	NA	Sato et al. (2003)
QT3031	18662636	c.436G>A	p.Glu146Lys	probably damaging	0	Hemi	hetero	The Retinoschisis Consortium (1998)
QT2964	18665414	c.223G>T	p.Glu75*	–	1/87544	Hemi	hetero	The Retinoschisis Consortium (1998)
QT3526	18665444	c.193T>C	p.Tyr65His	probably damaging	0	Hemi	hetero	Novel
QT3526	18665453	c.185‐1G>C	–	splicing change	0	Hemi	hetero	The Retinoschisis Consortium (1998)
QT3335	18660219	c.579dup	p.Ile194fs	frameshift	0	Hemi	hetero	The Retinoschisis Consortium (1998)
QT3508	18662607	c.465C>G	p.Tyr155*	–	0	Hemi	hetero	Novel
QT3139	18660200	c.599G>A	p.Arg200His	probably damaging	0	Hemi	hetero	The Retinoschisis Consortium (1998)
QT3108	18662650	c.422G>C	p.Arg141Pro	probably damaging	0	Hemi	hetero	Novel
QT3463	18665450	c.187T>C	p.Cys63Arg	probably damaging	0	Hemi	hetero	Novel
QT3523	18674849	c.108del	p.Ala37fs	frameshift	0	Hemi	NA	Novel

Abbreviations: hemi, hemizygous; hetero, heterozygous; NA, not available.

### 3.2 Clinical data

**Figure 3:**
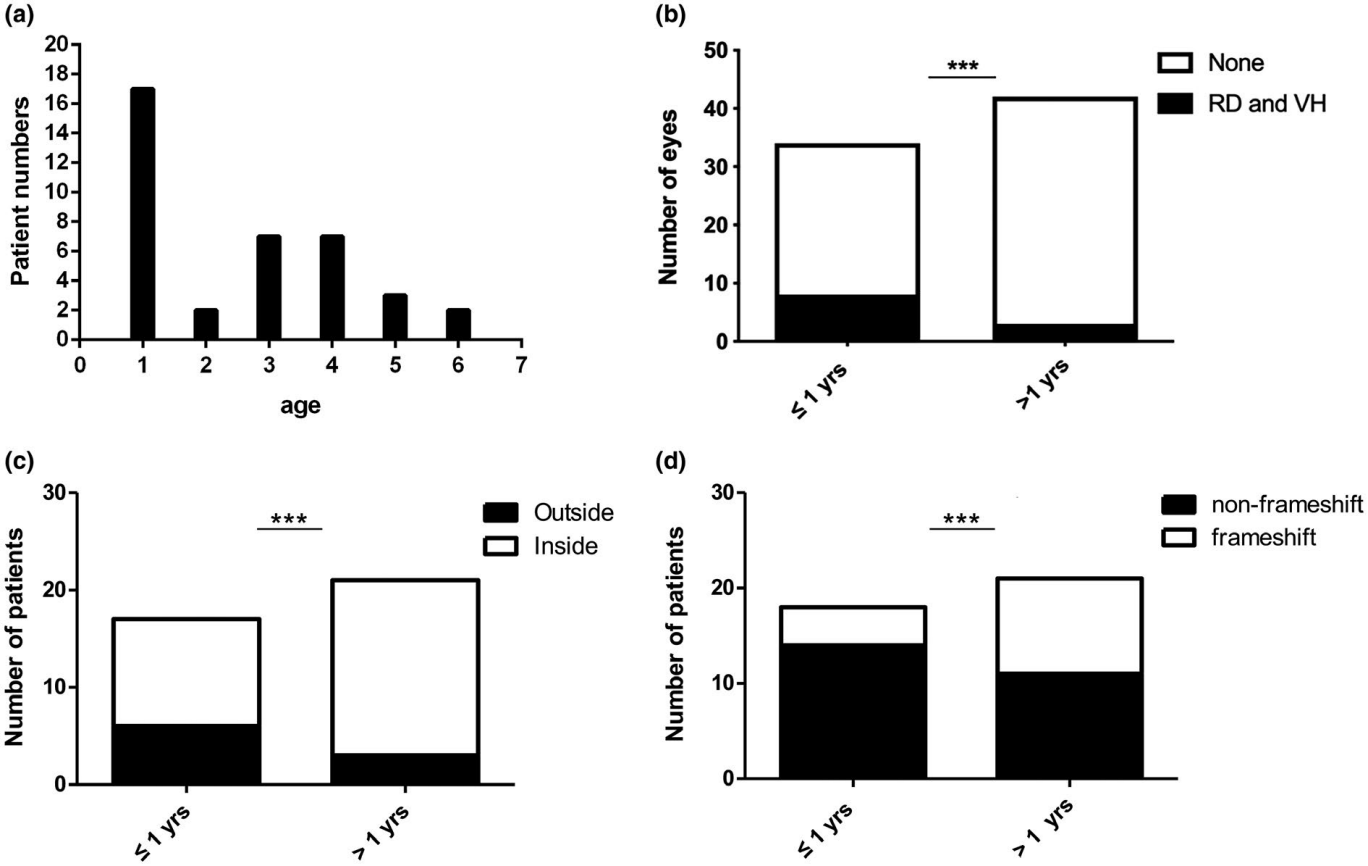
Clinical manifestations. (a) The distribution of the onset age. (b) The distribution of complications between groups (onset age ≤1 and >1 year old) with *p* < 0.001. (c) The mutation locations between groups (*p* < 0.001). (d) The mutation types between groups. *** *p* < 0.001

**Figure 4:**
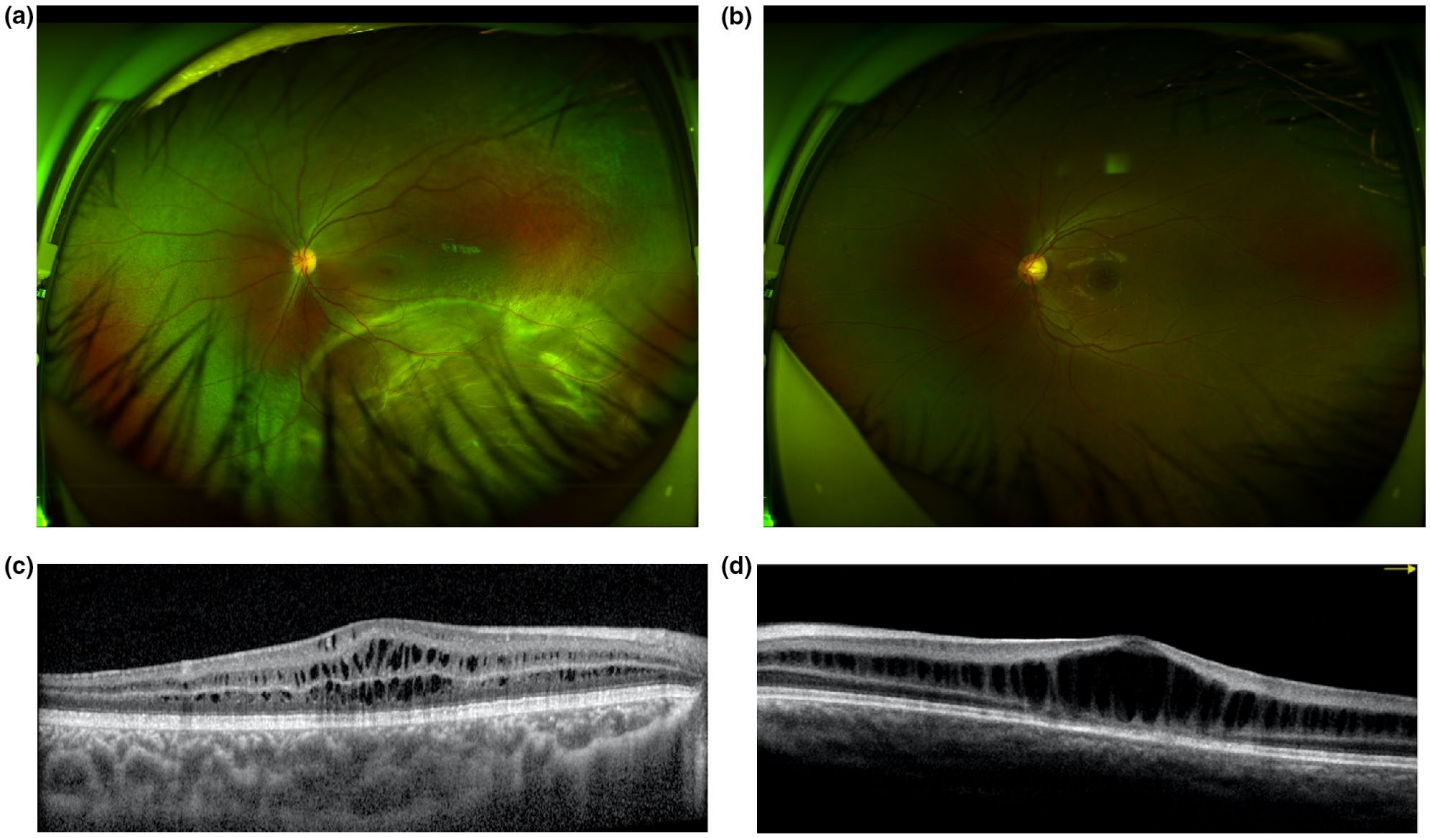
The scanning laser ophthalmoscope (SLO) and OCT of XLRS patients. (a) SLO showing macular and periphery involvement of the retina. (b) SLO showing only the macular involvement. (c) OCT showing inner nuclear layer and outer nuclear layer splitting. (d) OCT showing inner nuclear layer splitting

All of the patients were male, with a median examination age of 4 years old (range 0.1–21 years) and a median onset age of 2 years old (range 0.1–6 years) including 17 patients with the onset age equal to or less than 1 year old and 21 patients with the onset age more than 1 year old (Table 2, Figure 3a). The first symptoms consisted of no following (5/38, 13.15%), poor vision (27/38, 71.05%), and strabismus (6/38, 15.79%). The BCVA examinations were conducted in patients more than 4 years who could cooperate with the examination, and the results ranged from no light perception to 0.60 (Table 2). FP was available for 66 eyes of 33 probands, and a spoke‐wheel pattern combined peripheral RS was detected in 51 (77.27%) eyes (Figure 4a,b). OCT was available for 28 probands, from which inner nuclear layer RS was detected in 13 (46.43%) probands (Figure 4c,d) and inner nuclear layer RS combined with outer nuclear layer RS was detected in 15 (53.57%) probands. ERG was obtained for 18 probands and showed an electronegative waveform pattern with a b‐wave amplitude smaller than that of an a‐wave. Nine probands had complications, including five patients (seven eyes) with retinal detachments, and four patients (four eyes) with vitreous hemorrhages.

**Table 2:** Clinical data of probands with *RS1* mutations

Family ID	DNA change	Age at	Age at	First	BCVA		FP		OCT		Complications	
		E(y)	Onset (y)	Symptoms	OD	OS	OD	OS	OD	OS	OD	OS
QT2172	c.175T>G	5	FMB	PV	–	–	SWM	SWM	IN/ONRS	IN/ONRS	–	–
QT2173	c.647T>C	5	4	PV	–	–	–	–	IN/ONRS	IN/ONRS	–	–
QT2294	c.305G>A	3	3	PV	–	–	SWM, PR	SWM, PR	–	–	–	RD
QT2312	c.608C>T	3	1	PV	–	–	SWM, PR	SWM, PR	INRS	INRS	–	VH
QT2325	c.587C>T	0.8	0.1	NF	–	–	RD	RD	–	–	RD	RD
QT2329	c.26T>A	8	FMB	PV	0.04	NLP	SWM, PR	SWM, PR	INRS	INRS	–	RD
QT2347	c.391A>C	0.1	0.1	NF	–	–	SWM	SWM	IN/ONRS	IN/ONRS	–	–
QT2361	c.276G>C	4	FMB	NF	–	–	SWM, PR	SWM, PR	IN/ONRS	IN/ONRS	–	–
QT2390	c.540_675+682del	5	4	Strabismus	0.15	FC/30CM	–	–	INRS	INRS	–	–
F1	c.305G>A	5	3	PV	0.25	0.12	SWM, PR	SWM, PR	IN/ONRS	IN/ONRS, MH	–	–
F2	c.643G>A	5	4	PV	0.15	0.25	SWM	SWM	IN/ONRS	IN/ONRS	–	–
F3	c.305G>A	0.6	0.1	NF	–	–	SWM, PR	SWM, PR	–	–	RD	RD
F4	c.98G>A	4	1	PV	0.40	FC/40CM	SWM, PR	SWM, PR, MH	INRS	INRS, MH	–	–
F5	c.214G>A	4	4	PV	0.03	0.02	SWM, PR	SWM, PR	–	–	–	–
F6	c.305G>A	2	1	Strabismus	–	–	PR	PR	–	–	–	–
QT2462	c.575C>A	9	4	PV	0.10	0.30	–	–	INRS	INRS	–	–
QT2571	c.637C>T	5	4	PV	0.20	0.20	SWM, PR	SWM, PR	INRS	INRS	–	–
QT2590	c.138dup	7	1	PV	0.20	0.60	SWM, PR	SWM, PR	INRS	INRS	–	–
QT2650	c.214G>A	1	1	Strabismus	–	–	–	–	–	–	–	–
QT2681	c.52+5G>A	3	3	PV	FC/30CM	0.40	SWM, PR	SWM, PR	IN/ONRS	Normal	–	–
QT2905	c.592_600del	4	3	PV	0.40	0.05	SWM, PR	SWM, PR	IN/ONRS	IN/ONRS	–	–
QT2765	c.579dup	4	2	PV	–	–	SWM, PR	SWM, PR	IN/ONRS	IN/ONRS	–	–
QT2848	c.667T>C	10	5	PV	0.20	0.25	SWM, PR	SWM, PR	INRS	INRS	VH	–
QT3065	c.587C>T	1	1	Strabismus	–	–	SWM, PR	SWM, PR	–	–	–	–
QT3046	c.214G>C	7	6	PV	0.10	0.10	SWM, PR	SWM, PR	IN/ONRS	IN/ONRS	–	–
QT3032	c.579dup	6	3	PV	HM/30CM	0.10	SWM, PR	SWM, PR	INRS	INRS	–	–
QT2337	c.187T>C	5	3	PV	0.20	0.01	SWM, PR	SWM, PR	INRS	INRS	–	–
QT3042	c.590G>A	6	5	PV		NA	–	–	IN/ONRS	IN/ONRS	–	–
QT2931	c.522+1G>A	21	3	PV	0.02	0.10	SWM, PR	SWM, PR	INRS	INRS	–	–
QT3031	c.436G>A	2	1	PV	NA	NA	SWM, PR	SWM, PR	–	–	–	–
QT2964	c.223G>T	4	2	PV	0.20	0.20	SWM, PR	SWM, PR	INRS	INRS	–	–
QT3526	c.193T>C	4	1	Strabismus	0.25	0.10	SWM, PR	SWM, PR	INRS	INRS	–	VH
QT3526	c.185‐1G>C	1	1	Strabismus	0.25	0.10	SWM, PR	SWM, PR	INRS	INRS	–	VH
QT3335	c.579dup	11	6	PV	0.40	0.30	SWM	SWM	IN/ONRS	IN/ONRS	–	–
QT3508	c.465C>G	13	5	PV	NA	NA	SWM	SWM	–	–	–	–
QT3139	c.599G>A	1	0.1	Strabismus	NA	NA	RD	SWM, PR	INRS	INRS	RD	–
QT3108	c.422G>C	2	1	PV	–	–	SWM, PR	SWM, PR	–	–	–	–
QT3463	c.187T>C	3	1	PV	–	–	PR	PR	–	–	–	–
QT3523	c.108del	6	4	PV	0.20	0.20	SWM, PR	SWM, PR	INRS	INRS	–	–

Abbreviations: BCVA, best corrected visual acuity; E, examination; FC, finger counting; FMB, first few months after birth; FP, fundus photography; IN/ONRS, inner nuclear layer and outer nuclear layer retinoschisis; INRS, inner nuclear layer retinoschisis; NA, not available; NF, no following; OCT, optical coherence tomography; OD, right eye; OS, left eye; PR, peripheral retinoschisis; PV, poor vision; RD, retinal detachment; SWM, spoke‐wheel pattern in the macula; VH, vitreous hemorrhage; y, years old.

### 3.3 Difference between patients with onset age equal to or less than 1 year old and more than 1 year old

The patients with the onset age equal to or less than 1 year were assigned to group A, and the patients with the onset age more than 1 year old were assigned to group B. Retinal detachment and vitreous hemorrhage were considered to be severe complications of RS which were presented in more patients of group A than patients of group B (*p* < 0.001, Figure 3b) between groups. Significant difference was also detected in mutation locations (inside the discoidin domain and outside the discoidin domain) and mutation types (non‐frameshift mutations and frameshift mutation) (*p* < 0.001, Figure 3c,d). Patients in group A had more mutations outside the discoidin domain and had more non‐frameshift mutations.

### 3.4 Clinical data of proband with c.540_675+682del mutation

The patient (QT2390) with the gross deletion mutation (c.540_675+682del) was noticed to have strabismus at the age of 4 years. No treatment was applied to him over the next 1 year. At the age of 5, his BCVAs were 0.15 in the right eye and finger counting at 30 centimeters in the left eye. Macular ectopia in the left eye was showed in the fundus images, which was the reason for poor vision. And the poor vision contributed to the development of strabismus. Splitting of the inner nuclear layer was found in both eyes via OCT (Figure 2c).

### 3.5 Clinical data of probands with the same mutations

Similarity and differences were present in probands with the same mutations. RS was present in the macular area and peripheral retina in all the patients with the p.Arg102Gln mutation; however, retinal detachment also developed in two patients. Among the three patients with the c.579dup mutation, two patients had foveal and peripheral RS and one patient had foveal RS only. Two patients had inner nuclear layer RS combined outer nuclear layer RS, and one patient presented with inner nuclear layer RS.

### 3.6 Rare XLRS phenotypes

**Figure 5:**
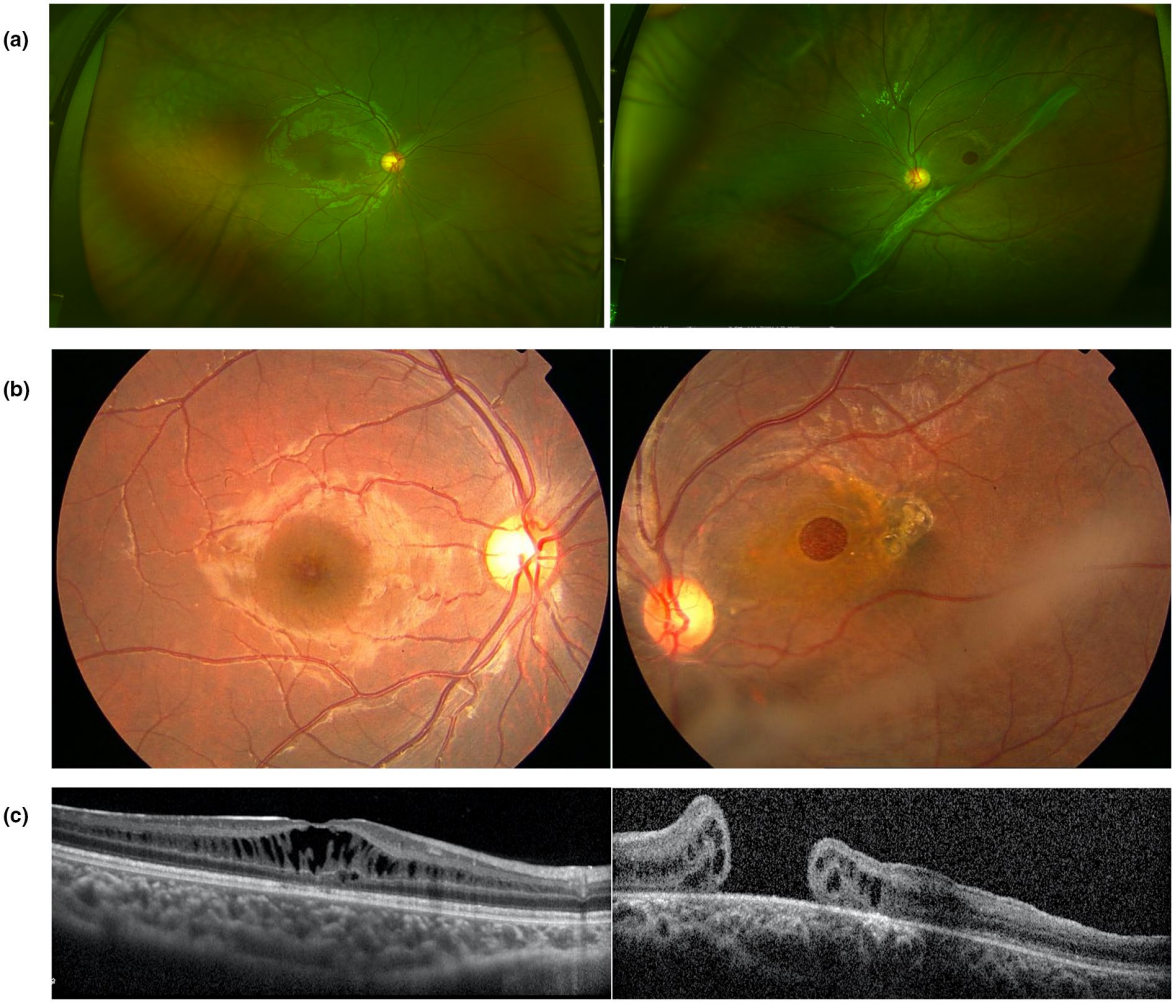
Clinical features of F4. The left column shows the examination of the right eye and the right column shows the examination of the left eye. (a) Scanning laser ophthalmoscope photography of F4. (b) FP of F4. (c) OCT of the right eye with retinoschisis. OCT of the left eye with macular hole (bottom line, right)

Clinical features varied even within the same patient. A rare phenotype was present in this study. A lamellar macular hole was present in one eye but absent in the contralateral eye in patient F1. A macular hole was present in the left eye but absent in the right eye in patient F4 as well as in patient QT2462 (Figure 5).

## 4 DISCUSSION

In this study, 38 early onset XLRS probands with a pathogenic *RS1* hemizygous mutation were included. Thirty different mutations were detected, including 16 known and 14 novel mutations. Mutation c.214G>A (p.Glu72Lys), c.305G>A (p.Arg102Gln) and c.579dup (p.Ile194fs) were considered to be the most common mutations in this cohort. This cohort emphasized the clinical manifestations with early onset XLRS. Foveal and peripheral RS was present in 77.27% of patients. Inner nuclear layer RS only was detected in 46.43% of patients, and inner nuclear layer RS combined with outer nuclear layer RS was detected in 53.57% of patients. Significant difference was detected in complications, mutation types, and mutation locations between patients less than or equal to 1 year old and old than 1 year old.

Phenotypes of this cohort showed differences from a previous study. Molday et al. mentioned that peripheral RS was present in fewer than 50% of the affected XLRS patients (Molday et al., 2012); however, peripheral RS was present in 77.27% of patients in the current study. Inner nuclear layer RS was detected in 46.43% of patients, and inner nuclear layer RS combined with outer nuclear layer RS was detected in 53.57% of patients. Retinal detachment and vitreous hemorrhages was present in approximately 5% of all affected males (Molday et al., 2012) in the general population. Another cohort demonstrated retinal detachment or severe macular lesion happened in 21.4% (3/14) before the age of 4 years, which was consistent with our study. The severe complication including retinal detachment and vitreous hemorrhages presented in 21.1% (8/38), especially in patients with the onset age less than 1 year old.

XLRS exhibits a high degree of phenotypic variability (Apaolaza et al., 2016). Several studies have illustrated that even among patients with the same mutations, there is still a great variation in disease severity (Eksandh et al., 2000; Pimenides et al., 2005; Xiao et al., 2016). Our study also demonstrated the clinical variability in XLRS patients but failed to find a phenotype‐genotype correlation. We summarized the clinical features of patients with the same mutation, differences in the severity of clinical phenotypes, extent of the retinal schisis, and complications. Additionally, even in the same patient, the ocular manifestation varied between the eyes. Some of the complications, such as a vitreous hemorrhage or retinal detachment, were detected in only one eye but absent in the contralateral eye. Overall, phenotypic variability makes it difficult to diagnose XLRS, especially in early onset patients who cannot cooperate with the ophthalmic examinations.

*RS1* mutations were detected by a clinical panel, and the panel contained 126 genes which was reported to be the common causative genes of the inherited retinal diseases from the Retinal Information Network (RetNet: https://sph.uth.edu/retnet/). Although other variations have also been detected by clinical panel sequencing, however, after multiple bioinformatic analysis of the sequencing data, mutation in *RS1* was the only mutation which we considered to be the reason for the XLRS.

In this study, genetic causes and clinical features of 38 Chinese probands with early onset XLRS were illustrated. The 14 novel mutations presented in this study expanded the mutation spectrum of *RS1*. Compared to the previous reported cohort of XLRS, patients with early onset age had higher incidence of peripheral RS as well as higher incidence of inner and outer nuclear layer splitting. Among the early onset cohort with RS, the patients with the onset age less than or equal to 1 year old were more likely to develop severe complications. Meanwhile, the *RS1* mutation types and mutation locations were different. With an understanding of early onset XLRS, early diagnosis, and close observation may reduce the vision threatening complications.

## CONFLICT OF INTEREST

All authors have no conflict of interest in the conduct of this study.

## AUTHOR CONTRIBUTIONS

*Data duration*: Li Huang, Zhirong Wang, Xiaoling Luo, Wenmin Sun, Panfeng Wang, and Chonglin Chen. *Funding acquisition*: Li Huang and Xiaoyan Ding. *Investigation*: Xiaoyan Ding. *Writing – original draft*: Li Huang. *Writing – review & editing*: Limei Sun and Xiaoyan Ding.

## Data Availability

The online sources used in this study was as follows. HGMD: http://www.hgmd.cf.ac.uk/ac/index.php Retinal Information Network (RetNet): https://sph.uth.edu/retnet/ Human Genome Variation Society (HGVS): http://www.hgvs.org The Exome Aggregation Consortium (ExAC): http://exac.broadinstitute.org.
